# Integrated genomic and clinical indicators for predicting foetal chromosomal abnormalities: development and validation of a nomogram model

**DOI:** 10.7189/jogh.16.04092

**Published:** 2026-04-10

**Authors:** Yingjie Zhou, Peng Liu, Xiaolin Zhang, Qiaoling Geng, Hanbing Jia, Zi Wang, Xiaojing Zhou, Haiyan Li, Jia Zhang, Ci Liu, Xuejun Liu, Weijun Kang

**Affiliations:** 1 School of Public Health, Hebei Medical University, Shijiazhuang, China; 2 Hebei Reproductive Health Hospital, Hebei Key Laboratory of Reproductive Medicine, Shijiazhuang, China

## Abstract

**Background:**

Foetal chromosomal abnormalities are identified as one of the leading causes of adverse pregnancy outcome in pregnancies that are referred on suspicion of structural abnormalities in the foetus. While various types of genomic analyses can be carried out in order to determine prenatal risk factors, no single nomogram in existence combines these factors to provide the probability of chromosomal abnormalities in the foetus.

**Methods:**

This was a retrospective cohort analysis among pregnant women referred for possible foetal structural anomalies who received invasive prenatal diagnostic tests at two tertiary referral centres within Hebei Province. The study participants received uniform prenatal evaluation and genomic analysis that included non-invasive prenatal testing as well as copy number variation analysis. Foetal anomalies identified by ultrasound were re-evaluated by expert reviewers, with confirmed structural anomalies being candidate predictors. Predictors were assessed with multivariable logistic regression analysis. Performance of the models was assessed by the discrimination method, calibration with bootstrap resampling, decision curve analysis, and internal validation with repeated cross-validation.

**Results:**

Abnormal results from Down syndrome screening, non-invasive prenatal testing, copy number variation sequencing, the presence of foetal structural abnormalities confirmed by ultrasound, and high gravidity were found to be independently associated with foetal chromosomal abnormalities. The nomogram showed high discriminative power, as evidenced by the area under the curve in the receiver operating characteristic curve graph, which exceeded 0.90. Calibration plots demonstrated good agreement between predicted and observed risks. Results from decision curve analysis suggested significant clinical benefit over a broad range of threshold probabilities. Validation results confirmed the stability of the nomogram.

**Conclusions:**

The proposed nomogram incorporates genomic technologies into clinical and ultrasound indicators for the precise estimation of risk on an individual basis for foetal chromosomal abnormalities. This tool may support prenatal counselling and assist clinicians in optimising diagnostic decision-making in pregnancies with suspected foetal structural abnormalities.

Foetal malformations are relatively common and complex issues in perinatal medicine. Though multiple factors are present, genetic causes predominate. Correct identification of such causes is important regarding the prognosis, genetic counselling, and reproductive planning, and still presents one of the great challenges in the care of the perinatal period.

Current approaches – ultrasound scanning, serological screening, and standard chromosomal karyotyping [1] – have notable limitations [2]. Ultrasound is essential for phenotypic evaluation but is operator- and gestational age-dependent and may miss subtle, transient, or non-phenotypic anomalies, as well as underlying genetic mechanisms [3]. Serum screening mainly detects common aneuploidies and provides limited information on sub chromosomal or monogenic causes. Even in foetuses with normal karyotypes, clinically relevant genetic abnormalities, such as pathogenic copy number variations (CNVs) and single-gene variants, often remain undetected [4]. These limitations challenge timely risk assessment, genomic workup, and counselling.

Due to the fast emergence of next-generation sequencing, these kinds of hurdles have been overcome, opening new avenues for making the diagnosis. Some of the clinical uses of the genomic technology include non-invasive prenatal screening for common aneuploidies [5,6], genome-wide screening for pathological copy number variations by CNV sequencing [7], and exome sequencing in supposed monogenetic disorders [8]. Long-read sequencing may add to the ability to identify complex abnormalities in selected patients. Each technique, however, has its limitations with respect to the subject, biological limitations, interpretability, and ability to detect. Hence, its clinical adaptation, incorporating phenotype and genomic information, is the key to improve risk assessment for the genetic defect in the structurally abnormal foetuses.

Despite the progress in the genomic area, the problem of the translational gap in the area of prenatal care for structural abnormalities in the foetus has persisted. There is a need for a validation of models, and most of these models have been single-modal and non-validated in the area of high-risk referrals, and the risk stratification has not been validated in terms of phenotypic variability. A nomogram was proposed that included non-invasive genomic data in addition to clinical data, with the exception of Whole-exome sequencing (WES) and long-read sequencing because of the small utility in the general population.

## METHODS

### Study population

In the retrospective cohort study, two affiliated tertiary care-referral hospitals (Hebei Province Prenatal Diagnosis Centre and Hebei Reproductive Health Hospital) were intended to participate in this study between June 2019–October 2024. Consecutive eligible pregnancies were intended to participate due to suspected foetal anomaly in invasive foetal analysis due to the possible bias which might arise because of selection. Data collection will continue up to the time the pregnancy is terminated or the birth occurs.

Inclusion criteria were as follows:

(1) eligible participants were pregnant women referred for suspected foetal structural abnormalities on screening ultrasonography during the study period and underwent invasive prenatal diagnostic testing

(2) voluntary participation with written informed consent.

Referral indication does not necessarily equal a confirmed anomaly; confirmation was determined by standardised expert re-evaluation at our centre, and confirmed structural abnormality was used as a predictor in the analyses.

Exclusion criteria were:

(1) pregnant women with severe comorbidities or pregnancy complications

(2) foetuses with known major chromosomal abnormalities (*e.g.* Down syndrome diagnosed through conventional methods).

To improve reproducibility and reduce confounding, we pre-specified candidate predictors based on clinical relevance and existing literature, and we considered potential confounders including maternal age, gestational age at sampling, gravidity/parity, and ultrasound phenotype; confounding was addressed through multivariable modelling with assessment of collinearity.

### Sample collection

Samples were obtained with informed consent as part of clinically indicated procedures: amniocentesis from 16–24 weeks, with chorionic villus sampling from 11–14 weeks performed under continuous ultrasound guidance. Samples were maintained under temperature regulation, with processing undertaken in a specialised laboratory. Additionally, 5–10 mL of blood, considered peripheral, was taken from the mother and partner as paired blood samples for DNA studies.

Comprehensive clinical and imaging data, including ultrasound and magnetic resonance imaging, were collected. ‘Ultrasound abnormality’ was defined as a foetal structural anomaly confirmed by expert assessment; unconfirmed findings were classified as normal. For subgroup analyses, findings were categorised by single- *vs.* multi-system involvement and major *vs.* isolated anomalies, used only for sensitivity analyses and not in the primary nomogram.

### Testing methods

#### Non-invasive prenatal testing (NIPT)

Non-invasive prenatal testing was performed according to standard massively parallel sequencing protocols. Sequencing reads were aligned to the human reference genome and Z-scores were calculated using a validated bioinformatics pipeline.

Abnormal maternal serum screening was defined as a calculated risk ≥1/270 for trisomy 21 and/or ≥1/350 for trisomy 18, based on routine first-trimester combined screening at 11–13 + 6 weeks’ gestation and/or second-trimester triple or quadruple screening at 15–20 + 6 weeks, with risk estimates generated using accredited laboratory software.

### CNV-seq analysis

Copy number variation sequencing was carried out using foetal DNA with quality-controlled libraries and sequencing on the Illumina platform. Raw-data files were aligned to GRCh37/hg19 using the Burrows-Wheeler Aligner (BWA), and the calling of the CNVs followed by deviation in read depth using the CNVnator algorithm. Variants were classified according to American College of Medical Genetics and Genomics (ACMG) /ClinGen recommendations, and pathogenic, likely pathogenic, and unknown-significance variants were considered abnormal and negative, respectively.

### WES

Whole-exome sequencing may be used within the clinical diagnostic workflow; however, because WES results are not a meaningful stand-alone cohort-level predictor and were not uniformly available for all participants, WES was not included as a candidate predictor in the nomogram modelling. Detailed laboratory procedures are provided in Supplementary Methods S1 in the **Online Supplementary Document**.

### Third-generation sequencing

Long-read third-generation sequencing may be used within the diagnostic workflow; however, because these results are not meaningful stand-alone cohort-level predictors and were not uniformly available for all participants, they were not included as candidate predictors in the nomogram modelling. Detailed procedures are provided in Supplementary Methods S2 in the **Online Supplementary Document**.

### Statistical analysis

Statistical analyses were carried out in *R*, version 4.3.2 (R Foundation for Statistical Computing, Vienna, Austria). Normality of continuous variables was checked by Shapiro-Wilk test and expressed as mean ± standard deviation or median (interquartile range). Comparisons between groups of normally distributed variables used *t* test or Wilcoxon rank-sum test. Categorical variables are expressed as number (%). Comparisons between groups are carried out by χ^2^ test or Fisher exact test. *P*-value ≤0.05 was taken as significant.

The selection of predictors occurred through an objective and repeatable method. Candidate variables were selected *a priori* based on relevance to clinical practice and previous research. Univariate logistic regression analysis selected variables which related to foetal chromosomal abnormalities with *P* < 0.10 significance, or those of prime importance to inclusion to multivariate analysis. Backward elimination on Akaike Information Criterion defined variables to retain within models, presenting effect measures as odds ratio (OR) with 95% confidence interval (CI). The variance inflation test is concerned with multivariance. A variance inflation factor (VIF)<5 is generally acceptable.

Continuous predictors (*e.g.* maternal age and gravidity) were retained to preserve information. Linearity in the logit was assessed with restricted cubic splines; linear terms were used when no significant nonlinearity was detected. Model discrimination was evaluated using receiver operating characteristic (ROC) curves and area under the curve (AUC), while calibration was assessed via 1000-bootstrap resampling and calibration plots. A nomogram was constructed from the final multivariable model using the rms package (version 6.8-1) in *R*, version 4.3.2 (R Foundation for Statistical Computing, Vienna, Austria).

Internal validation was performed using 10-fold cross-validation repeated 100 times, with mean AUC reported. Clinical utility was assessed by decision curve analysis across thresholds of 2–60%. No missing values were present for candidate predictors or final model variables (Down syndrome screening, NIPT, ultrasound findings, CNV status, and gravidity), so a complete-case analysis was applied. Analyses were conducted in *R*, version 4.3.2 (R Foundation for Statistical Computing, Vienna, Austria) using the rms package (version 6.8-1) for nomogram construction and calibration, the pROC package (version 1.18.5) for ROC analysis, the rmda package (version 1.6) for decision curve analysis, and the car package (version 3.1-2) for collinearity assessment.

## RESULTS

### Participant characteristics and pregnancy outcomes

In all, 674 women were evaluated, 619 (91.8%) with a normal karyotype and 55 (8.2%) with an abnormal karyotype. Maternal age mean values were 28.4 ± 5.1 years, with a non-significant difference when evaluated by karyotype abnormality (*P* = 0.053); there were no differences for paternal age (*P* = 0.11). Gravidity differed significantly between the groups, with a higher proportion of women having gravidity ≥4 in the abnormal karyotype group than in the normal karyotype group (39% *vs*. 11%, *P* = 0.004); similarly, parity also differed significantly, with a higher proportion of women having ≥2 live births in the abnormal karyotype group than in the normal karyotype group (15% *vs*. 6%, *P* = 0.022). Surrogate markers were also evaluated; there were equal numbers of foetuses (*P* > 0.9).

Ultrasound abnormalities were more frequent in the chromosomal abnormality group (64 *vs*. 10%; *P* < 0.001), as were magnetic resonance imaging abnormalities (35 *vs*. 6%; *P* < 0.001) and high-risk findings on transthoracic ultrasound (60 *vs.* 2%; *P* < 0.001). Abnormal NIPT (29 *vs.* 4%; *P* < 0.001) and CNV findings (56 *vs*. 11%; *P* < 0.001) were also higher in this group. Some participants in the normal karyotype group had no confirmed anomalies on standardised re-evaluation, reflecting the referral-based enrolment criteria.

No significant intergroup differences were observed for other clinical parameters, including abnormal nuchal translucency (15 *vs.* 9%; *P* = 0.3), obstetric complications (24 *vs.* 19%; *P* = 0.5), conception via in vitro fertilisation (4 *vs.* 5%; *P* = 0.8), regular menses (96 *vs*. 97%; *P* > 0.9), or radiation exposure (0 *vs*. 1%; *P* > 0.9).

Study participants were recruited from the Hebei Prenatal Diagnosis Centre, Second Hospital of Hebei Medical University, and the Hebei Reproductive Health Centre. Nearly all participants (n/N = 671/674) were ethnic Han, representing a relatively homogeneous population (**Table 1**).

**Table 1:** Baseline characteristics table

Characteristics	Overall, n (%) N = 674	Normal karyotype, n (%) N = 619	Abnormal karyotype, n (%) N = 55	*P*-value
Age				0.053
*Mean (SD)*	28.4 ± 5.1	28.3 ± 5.1	29.7 ± 4.8	
Father Age				0.110
*Mean (SD)*	29.3 ± 4.5	29.2 ± 4.5	30.3 ± 4.4	
Gravidity				0.004
*1*	239 (35)	225 (36)	14 (25)	
*2*	186 (28)	177 (29)	9 (16)	
*3*	140 (21)	126 (20)	14 (25)	
*4*	84 (12)	68 (11)	16 (29)	
*5*	19 (3)	17 (3)	2 (4)	
*6*	4 (1)	4 (1)	0 (0)	
*7*	2 (0)	2 (0)	0 (0)	
Parity				0.022
*0*	307 (46)	284 (46)	23 (42)	
*1*	322 (48)	298 (48)	24 (44)	
*2*	40 (6)	34 (5)	6 (11)	
*3*	5 (1)	3 (0)	2 (4)	
Foetus number				>0.9
*1*	664 (99)	610 (99)	54 (98)	
*2*	9 (1)	8 (1)	1 (2)	
*3*	1 (0)	1 (0)	0 (0)	
NT				0.3
*Normal*	608 (90)	561 (91)	47 (85)	
*Abnormal*	66 (10)	58 (9)	8 (15)	
Ethnicity				>0.9
*Han Chinese*	671 (100)	616 (100)	55 (100)	
*Other ethnic groups*	3 (0)	3 (0)	0 (0)	
Regular menstruation				>0.9
*Abnormal*	20 (3)	18 (3)	2 (4)	
*Normal*	654 (97)	601 (97)	53 (96)	
IVF				0.8
*No*	638 (95)	585 (95)	53 (96)	
*Yes*	36 (5)	34 (5)	2 (4)	
USG				<0.001
*Normal*	575 (85)	555 (90)	20 (36)	
*Abnormal*	99 (15)	64 (10)	35 (64)	
MRI				<0.001
*Normal*	617 (92)	581 (94)	36 (65)	
*Abnormal*	57 (8)	38 (6)	19 (35)	
Down syndrome screening				<0.001
*Normal*	629 (93)	607 (98)	22 (40)	
*Abnormal*	45 (7)	12 (2)	33 (60)	
NIPT				<0.001
*Normal*	635 (94)	596 (96)	39 (71)	
*Abnormal*	39 (6)	23 (4)	16 (29)	
Bad pregnancy history				0.5
*Normal*	544 (81)	502 (81)	42 (76)	
*Abnormal*	130 (19)	117 (19)	13 (24)	
Radiation exposure				>0.9
*No*	667 (99)	612 (99)	55 (100)	
*Yes*	7 (1)	7 (1)	0 (0)	
CNV				<0.001
*Normal*	578 (86)	554 (89)	24 (44)	
*Abnormal*	96 (14)	65 (11)	31 (56)	

CNV – copy number variation, IVF – in vitro fertilisation, MRI – magnetic resonance imaging, NIPT – non-invasive prenatal testing, NT – nuchal translucency, SD – standard deviation, USG – ultrasonography

### Development and performance of the predictive model

Binary logistic regression was used to assess associations between ultrasound findings, Down syndrome screening, NIPT, CNV results, gravidity, and foetal chromosomal abnormalities, with categorical variables coded as in **Table 2**. The overall model was statistically significant, and all included variables were independent predictors (all *P* < 0.05) (**Table 3**).

**Table 2:** Defines the assignment rules for each variable in the study for quantitative analysis

Variables	Assignment scenarios	
USG	X1	0 = Normal;1 = Abnormal
Down syndrome screening	X2	0 = Normal;1 = Abnormal
NIPT	X3	0 = Normal;1 = Abnormal
CNV	X4	0 = Normal;1 = Abnormal
Gravidity	X5	Continuous (per one-pregnancy increase)
Pregnancy outcome	Y	0 = Normal karyotype; 1 = Abnormal karyotype

CNV – copy number variation, NIPT – non-invasive prenatal testing, USG – ultrasonography

**Table 3:** Logistic regression results for pregnancy outcomes

Variables	β	SE	Wald χ2	*P-*value	OR	95% CI
USG	1.852	0.470	15.538	<0.001	6.374	2.538, 16.012
Down syndrome screening	4.113	0.517	63.298	<0.001	61.117	22.189, 168.338
NIPT	2.722	0.552	24.326	<0.001	15.206	5.156, 44.848
CNV	1.852	0.476	15.164	<0.001	6.375	2.509, 16.195
Gravidity	0.401	0.180	4.933	0.026	1.493	1.048, 2.127
Constant	−5.823	0.655	79.030	<0.001	0.003	

CI – confidence interval, CNV – copy number variation, NIPT – non-invasive prenatal testing, OR – odds ratio, SE – standard error, USG – ultrasonography

Abnormal Down syndrome screening showed the strongest association with foetal chromosomal abnormalities (OR = 61.117; 95% CI = 22.189, 168.338). Non-invasive prenatal testing was also a significant predictor (OR = 15.206; 95% CI = 5.156, 44.848), while ultrasound (OR = 6.374; 95% CI = 2.538, 16.012) and CNV (OR = 6.375; 95% CI = 2.509, 16.195, respectively) abnormalities had similar effects. Primigravidity was associated with increased risk (OR = 1.493; 95% CI = 1.048, 2.127). No multicollinearity was detected (all VIFs ≈ 1, < 5) (Table S1 in the **Online Supplementary Document**).

**Figure 1:**
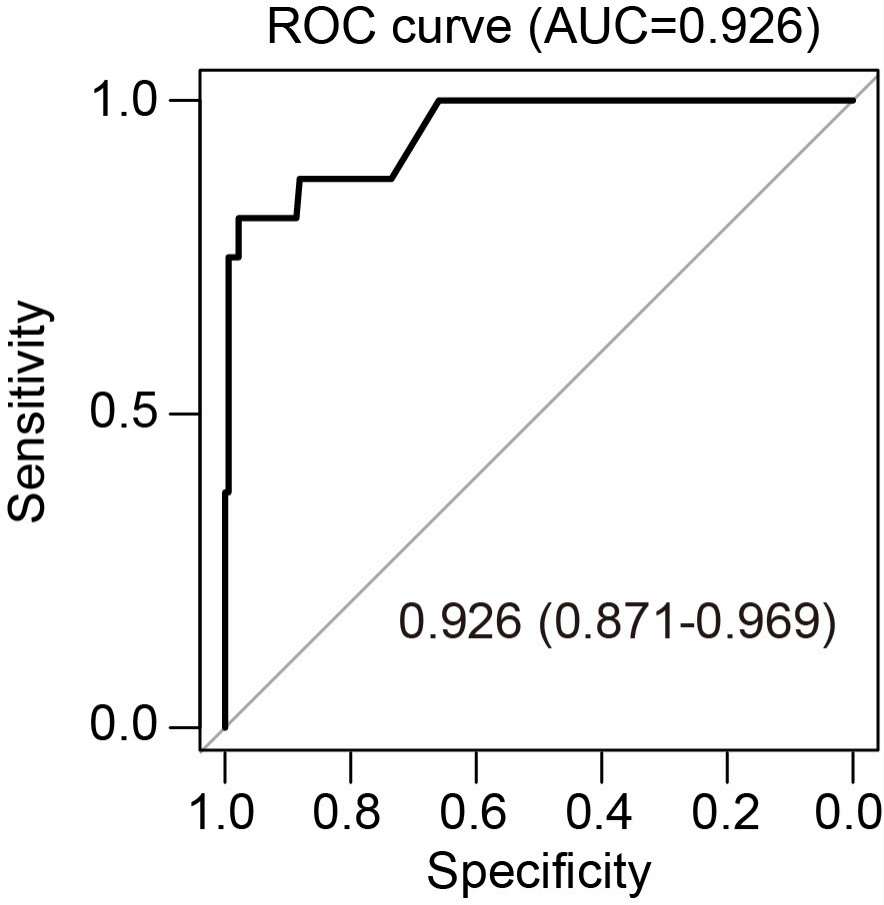
Receiver operating characteristic curve of the predictive model for foetal chromosomal abnormalities.

A multivariable logistic regression model was developed to predict abnormal foetal karyotype using first-trimester serum screening for trisomy 21/18, NIPT, ultrasound anomalies, CNV, and gravidity. The model showed high discrimination (C-index, ROC AUC = 0.926) (**Figure 1**). At the optimal cut-off of 0.287 (Youden index), sensitivity was 81.8%, specificity 97.9%, positive predictive value 77.6%, and negative predictive value 98.4% (Table S2 in the **Online Supplementary Document**).

Model stability and generalisability were evaluated using 10-fold cross-validation, yielding an AUC = 0.931, consistent with the apparent performance and confirming model robustness.

### Nomogram development

**Figure 2:**
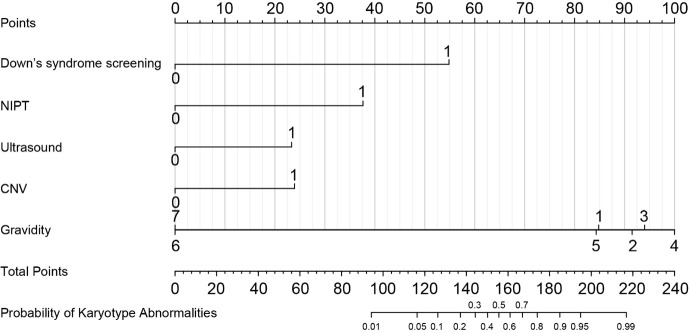
Nomogram for individualised prediction of foetal chromosomal abnormalities. Points are assigned to each predictor and summed to estimate the predicted probability.

This nomogram was specifically made to quantify the risk of chromosomal abnormalities in the foetus through the incorporation of essential clinical predictors found in maternity care, such as first-trimester screening for Down syndrome 21/18, NIPT, ultrasound findings, CNV analysis, and gravidity. **Figure 2** highlights the entirety of the predictor variables with their corresponding points.

### Calibration of the nomogram model

**Figure 3:**
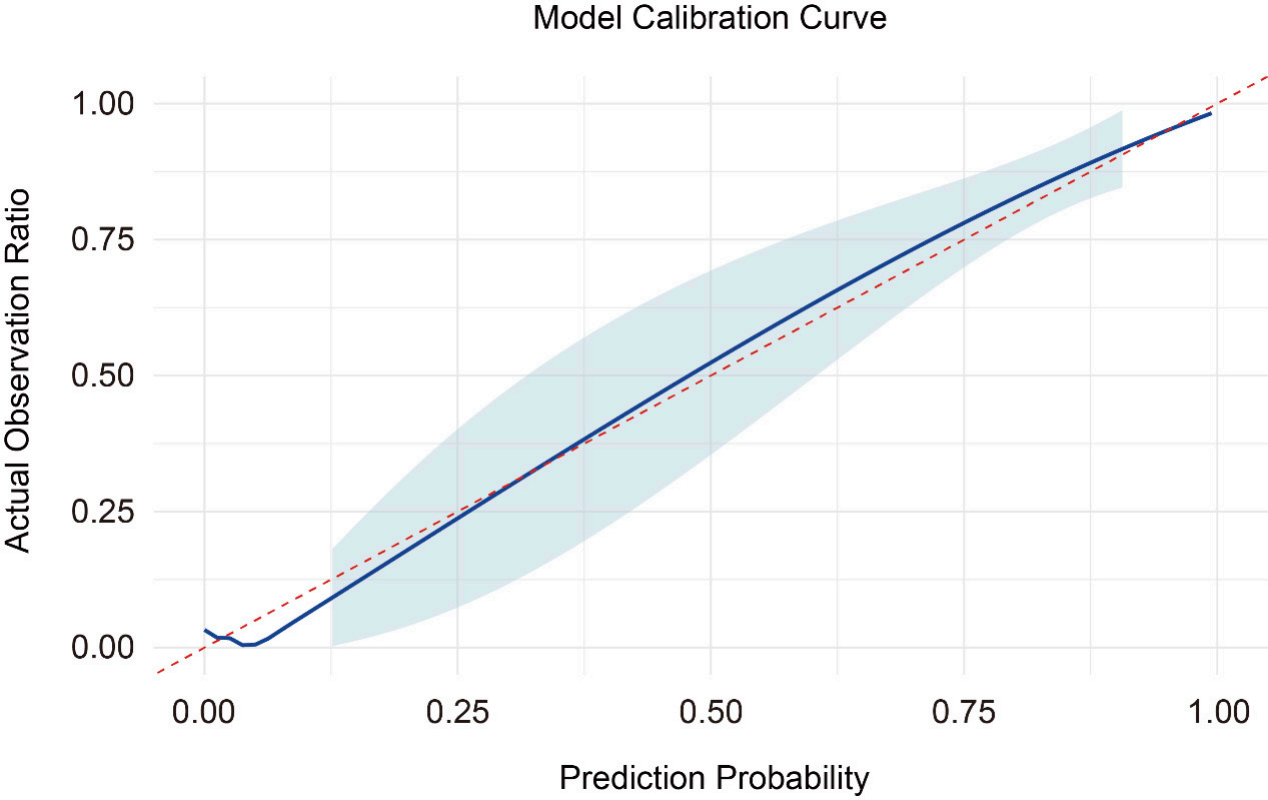
Calibration curve of the nomogram model.

A calibration plot was employed to check agreement between predicted and observed probabilities of events (**Figure 3**). The nomogram demonstrates a very good level of agreement between predicted risks and incidence, especially in the lower to intermediate regions of risks, to demonstrate validity.

### Subgroup performance by ultrasound phenotype (isolated *vs.* multiple anomalies)

After performing subgroup analysis based on the ultrasound-defined phenotypes, the model performed well for foetuses with confirmed structural anomalies (AUC = 0.975), those with isolated anomalies (AUC = 0.990), those with multiple anomalies (AUC = 0.957), while not performing well for those without confirmed anomalies (AUC = 0.833). This suggests that the nomogram performs well on referred cases with confirmed foetal structural abnormalities (Figure S1–3 and Table S1–2 in the **Online Supplementary Document**).

### Clinical utility

Decision Curve Analysis was employed to assess the clinical validity of our nomogram. Net benefit, which is based on discrimination, is visually displayed as three decision curves in Figure S4 in the **Online Supplementary Document**.

In the decision curve analysis, the ‘Predictive Model’ represents use of the nomogram to guide decision-making, the ‘All’ strategy assumes all cases are high risk and require further genetic testing, and the ‘None’ strategy assumes all cases are low risk with no additional testing needed.

Decision curve analysis showed that across a clinically relevant high-risk threshold range of 2–60%, the ‘Predictive Model’ strategy provided greater net benefit than ‘All’ or ‘None’, indicating that using the nomogram to guide selective genetic testing is more advantageous. Operating characteristics at selected thresholds (2, 5, 10, 30, and 60%) are provided in Table S2 in the **Online Supplementary Document** for clinical interpretation.

## DISCUSSION

### Principal findings in relation to the study objectives

This research combined innovative genomic analyses with traditional clinical indicators to create a nomogram predicting cases of foetal chromosomal abnormalities. Five independent predictors emerged: serum screening for trisomy 21/18, non-invasive prenatal testing, ultrasound abnormalities, CNV results, and gravidity. The nomogram had good model performance with high specificity (99.5%) and moderate sensitivity of 62.5% and good area under receiver operating characteristic curve (AUC-ROC) = 0.926. Decision curve analysis verified that it has high net benefits for various values of threshold probabilities between 2–60%.

To identify potential multicollinearity as well as interaction between predictors, checks were also conducted. Variance inflation factor values were low for all variables (Table S3 in the **Online Supplementary Document**), indicating no issues of instability in the model parameters. Sensitivity analyses for an interaction between abnormal NIPT and ultrasound resulted in no improvement in model performance or discriminative ability and were also non-significant (OR = 2.12, Fisher exact test *P* = 0.060) (Table S4–5 in the **Online Supplementary Document**), confirming the validity of the simplified model with only main effects.

### Technology-specific roles in an integrated prenatal diagnostic strategy

These genomic technologies are supplementary in prenatal testing. Non-invasive prenatal testing allows for high specificity screening of common aneuploidies but is less useful when it comes to unusual or sub chromosomal anomalies. Copy number variation sequencing (CNV-seq) can identify significant microdeletions/duplications for clinical significance, although unusual CNVs can be difficult to explain. Trio WES can be helpful if karyotyping and CNV-seq are inconclusive, specifically for potential monogenic conditions, but can be replaced by third-generation long-read sequencing if the case is complex or unresolved, although largely based on availability.

### Why combining ultrasound and genomics improves discrimination

Such genomic tools are complementary to the prenatal screening. Non-invasive prenatal testing enables high specificity screening for common aneuploidies, although not so much in the case of rare or sub microscopic abnormalities. Copy number variation sequencing enables the detection of substantial microdeletions/duplications for clinical significance, although difficult for the case of abnormal CNVs. Trio WES would be beneficial in those instances where the traditional karyotyping analysis and CNV-seq have been inconclusive, particularly for possible monogenic disorders, although replaced by the third generation long-read sequencing, considering the complexity of the situation, although highly dependent on the facility.

### Mechanistic plausibility: CNVs and foetal malformations

Copy number variations may contribute to foetal malformations through gene dosage effects (haploinsufficiency or triplosensitivity) affecting cardiac, neurodevelopmental, and cranial patterning processes, and may reveal recessive variants or regulatory disruptions. Consistent with this, CNV abnormalities were enriched in the chromosomal abnormality group and were independent predictors in multivariable models (OR = 6.375), supporting CNV-seq testing in foetuses with suspected anomalies even when standard karyotyping is normal.

### Clinical interpretation of predictors

Among predictors, high-risk serum screening for trisomy 21/18 had the largest effect, reflecting enrichment of aneuploid pregnancies in a referred population. Non-invasive prenatal testing also showed strong discrimination (OR = 15.206), and confirmed ultrasound abnormalities were independently predictive (OR = 6.374), while gravidity remained significant (OR = 1.493), possibly reflecting cumulative reproductive history and maternal age–related risk. These findings align with prior studies: Liao et al. [9] reported improved detection with integrated serum and genomic testing (AUC = 0.91), Yin et al. [10] highlighted CNV-seq for microdeletion/microduplication detection (OR = 6.375), and Yang et al. [11] noted gravidity as a risk factor. Differences from prior reports were observed: NIPT was more predictive in our cohort than Huang et al. [12], and ultrasound effect size exceeded that in Chen et al. [13], likely due to variations in diagnostic criteria and case mix.

### Implications for practice and policy (high specificity *vs*. moderate sensitivity)

The high specificity (99.5%) prevents the occurrence of the ‘false positive’ result, while the moderate sensitivity (62.5%) gives the nomogram ‘rule-in’ rather than ‘rule-out’ status. Clinically suspected pregnancies may undergo evaluation as per guidelines, but the nomogram may assist in stratifying testing and counselling for genomic analysis. The decision curve analysis indicated the existence of the ‘net benefit’ for the risk stratification for thresholds from 2–60%.

### Implementation and economic considerations

Although the nomogram is an inexpensive decision-making aid, some variables (*e.g.* NIPT, CNV-seq) require genomic analyses with uncertain costs, reimbursement, and availability. If such analyses are common practice in tertiary care centres, the nomogram could assist with the staged escalation, with primary emphasis placed on comprehensive genomic analysis, parental studies, and genetic counselling for those with high risk, potentially optimising resource allocation. Cost-effectiveness analyses could not be performed, as costs have not been evaluated; this should be considered in future multicentre studies.

First, the cohort was recruited from two centres in one province, which reduces population heterogeneity. Second, the model did not stratify for the type of structural anomaly, which might influence the probability of chromosomal, microdeletion, or gene variants. Third, variants of uncertain significance (VUSs) identified by copy number variation sequencing were regarded as non-anomalies; future studies should explore the contribution of VUSs and parental inheritance. Fourth, WES and third-generation sequencing were performed in only a small number of cases, which limited the evaluation of their incremental diagnostic values. As such, the nomogram reflects mainly larger chromosomal abnormalities and CNVs, while single-gene variants and complex rearrangements were not fully evaluated. Future large multicentre studies with the extended application of WES and long-read sequencing will be required to further validate and improve the performance and clinical utility of the model.

Future studies could populate more samples by multicentre collaboration, as well as develop different models for subtypes. There may be differences in the prevalence of chromosomal abnormalities, screening rates, ultrasound criteria, or laboratory procedures that could lead to drift, so studies would require validation, including possible recalibration. It may be beneficial to include other genomic information, WES, third-generation sequencing, with artificial intelligence risk estimators.

## CONCLUSIONS

We have built and internally validated an integrative nomogram for the prediction of chromosomal abnormalities based on both genomic and clinical data. Our model has strong discriminatory ability and clinical practicality, which makes our approach valid for clinical application in prenatal care for risk classification. External validation is indicated for universality.

## Additional material


Online Supplementary Document
