# Supplementary material for: Integrated genomic and clinical indicators for predicting foetal chromosomal abnormalities: development and validation of a nomogram model
Source: J Glob Health. 2026 Apr 10;16:04092. doi: 10.7189/jogh.16.04092 (PMC13067297; doi:10.7189/jogh.16.04092)
Supplement: Online Supplementary Document [file jogh-16-04092-s001.pdf]

Supplement to: Zhou Y, Peng L, Zhang X, Geng Q, Jia H, Zi W, Zhou X, Li H, Jia Z, Ci Lx, Liu X, Kang W. Integrated genomic and clinical indicators for predicting foetal chromosomal abnormalities: development and validation of a nomogram model. J Glob Health. 2026;16:04092.

Supplementary methods S1. whole-exome sequencing (WES)

Fetal tissue and blood samples from parents were employed for the study. The exonic regions were enriched for next-generation sequencing with library preparation, quality of sequencing, and alignment assessed. Variants were picked, annotated with OMIM and ClinVar databases, and segregation study validation employed for predictions of pathogenicity.

Supplementary Methods S2. Third-generation long-read sequencing

In cases with normal karyotype and CNVseq, but high clinical suspicion for monogenic disorders, trio WES was conducted on fetal and parental DNA to prioritize pathogenic/likely pathogenic variants. When appropriate, third-generation long-read sequencing (Nanopore) was employed to identify structural genomic variation not captured by standard sequencing methods.High-quality fetal DNA was obtained for third-generation long-read sequencing, and specialized bioinformatics analysis thereof. Results from WES and third-generation sequencing were combined with second-generation sequencing to improve diagnostic power. Because these advanced approaches were used selectively, their outcomes were not included as predictors in the original nomogram, but instead contributed in a qualitative fashion to the diagnostic approach as a whole.

Supplementary Table S1. Collinearity diagnostics for final predictors (variance inflation factors, VIF).

Predictor	VIF
Serum screening for trisomy 21/18 result	1.207
NIPT result	1.053
Ultrasound abnormality	1.176
CNV status	1.114
Gravidity	1.015

Note: All VIFs were < 5, indicating no evidence of problematic multicollinearity in the final model.

Supplementary Table S2. Threshold sensitivity analysis illustrating trade-offs between sensitivity and specificity.

Strategy	Thresho	Sensitivit	Specificit	PPV	NPV	Predicte	False

	ld	y	y			d positive, n (%)	negatives, n (%)
Low threshold (2%)	0.020	87.3% (76.0-93.7)	72.7% (69.1-76.1)	22.1% (17.1-28.1)	98.5% (96.9-99.3)	217 (32.2%)	7 (12.7%)
Low-moderate threshold (5%)	0.050	87.3% (76.0-93.7)	86.9% (84.0-89.3)	37.2% (29.4-45.8)	98.7% (97.4-99.4)	129 (19.1%)	7 (12.7%)
Moderate threshold (10%)	0.100	81.8% (69.7-89.8)	94.5% (92.4-96.0)	57.0% (46.0-67.3)	98.3% (96.9-99.1)	79 (11.7%)	10 (18.2%)
High threshold (30%)	0.300	78.2% (65.6-87.1)	98.2% (96.8-99.0)	79.6% (67.1-88.2)	98.1% (96.6-98.9)	54 (8.0%)	12 (21.8%)
Very high threshold (60%)	0.600	65.5% (52.3-76.6)	99.2% (98.1-99.7)	87.8% (74.5-94.7)	97.0% (95.4-98.1)	41 (6.1%)	19 (34.5%)
Youden-optimal	0.287	81.8% (69.7-89.8)	97.9% (96.4-98.8)	77.6% (65.3-86.4)	98.4% (97.0-99.1)	58 (8.6%)	10 (18.2%)
High-sensitivity (Sens≥85%)	0.059	87.3% (76.0-93.7)	86.9% (84.0-89.3)	37.2% (29.4-45.8)	98.7% (97.4-99.4)	129 (19.1%)	7 (12.7%)
High-specificity (Spec≥99.5%)	0.720	52.7% (39.8-65.3)	99.5% (98.6-99.8)	90.6% (75.8-96.8)	96.0% (94.1-97.2)	32 (4.7%)	26 (47.3%)

Note: Metrics were computed on the full cohort (N=674). Predicted positive indicates cases exceeding the decision threshold and thus potentially triggering escalation of downstream genetic evaluation in a triage workflow.

Supplementary Table S3. Discrimination of the nomogram across ultrasound phenotype subgroups.

Subgroup	N	Abnormal karyotype, n (%)	AUC (*95% CI*)
Overall cohort	674	55 (8.2%)	0.926 (0.871-0.969)
No confirmed structural abnormality on expert ultrasound (USG=0)	575	20 (3.5%)	0.834 (0.707-0.943)

Confirmed structural abnormality on expert ultrasound (USG=1)	99	35 (35.4%)	0.976 (0.940-1.000)
— Isolated anomaly (within USG=1)	35	10 (28.6%)	0.996 (0.983-1.000)
— Multiple anomalies (within USG=1)	64	25 (39.1%)	0.965 (0.909-1.000)

Note: AUCs were calculated using predicted probabilities from the final multivariable logistic regression model (serum screening for trisomy 21/18, NIPT, confirmed ultrasound abnormality, CNV result, and Gravidity). *95% CI*s were obtained by bootstrap resampling (2,000 iterations). Within the USG=1 subgroup, isolated vs multiple anomalies were classified from ultrasound report text as an exploratory grouping (single vs ≥2 distinct structural findings).

Supplementary Table S4. Sensitivity analysis testing interaction between NIPT and ultrasound abnormality (NIPT × USG).

Term	*OR*	*95% CI*	*P value*
NIPT result	13.444	4.065–44.461	<0.001
Ultrasound abnormality	5.916	2.165–16.169	<0.001
NIPT × USG	1.934	0.134–27.822	0.628
Serum screening for trisomy 21/18 result	60.351	22.168–164.301	<0.001
CNV status	5.785	2.294–14.590	<0.001
Gravidity	1.345	0.685–2.641	0.389

Note: The interaction term (NIPT × USG) was not statistically significant (AIC = 177.07; AUC = 0.904), and inclusion of the interaction did not materially change the main-effect estimates.

Supplementary Table S5. Association between abnormal NIPT and confirmed ultrasound abnormality (2×2 table).

	USG abnormal (1)	USG normal (0)	Row total
NIPT abnormal (1)	10	29	39
NIPT normal (0)	89	546	635
Total	99	575	674

Note: *Odds ratio* = 2.12 (*95% CI*=1.00–4.49); Fisher's exact *P* = 0.060.

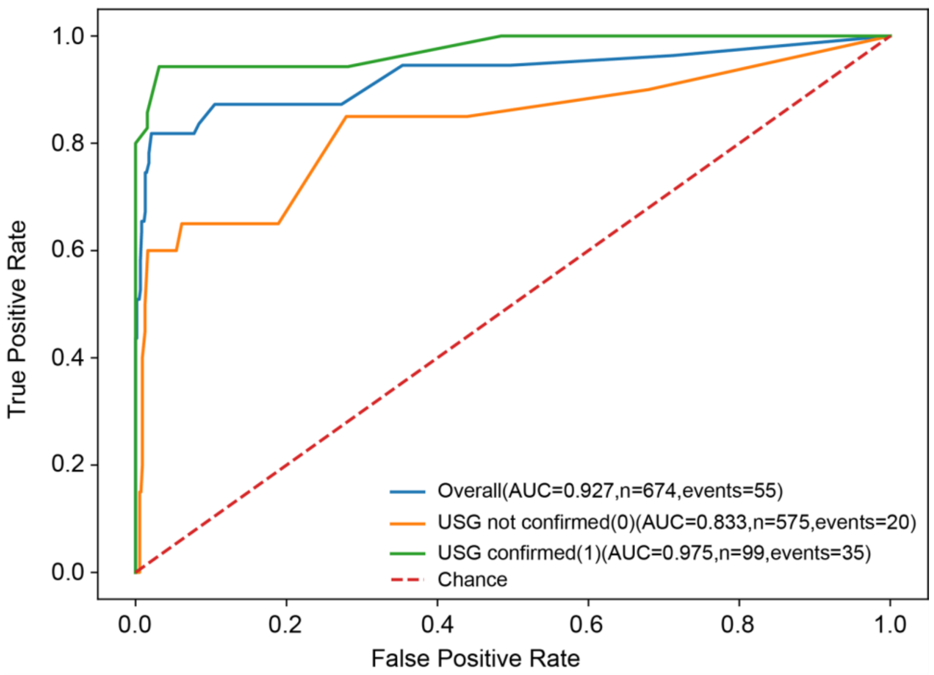

Supplementary Figure S1 ROC curves by ultrasound confirmation status

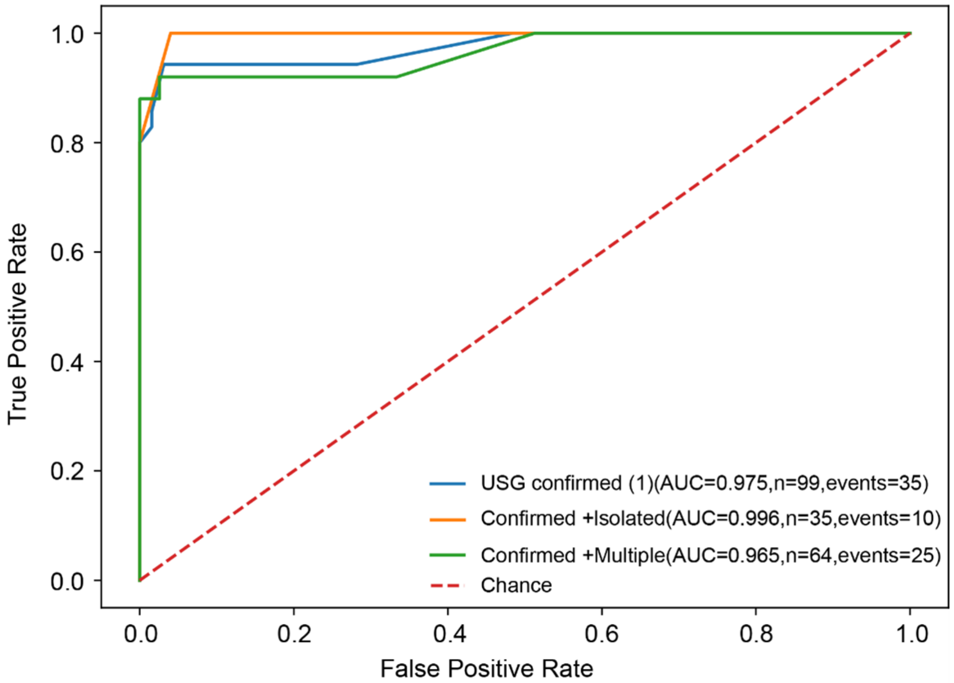

Supplementary Figure S2 ROC curves within confirmed structural anomalies

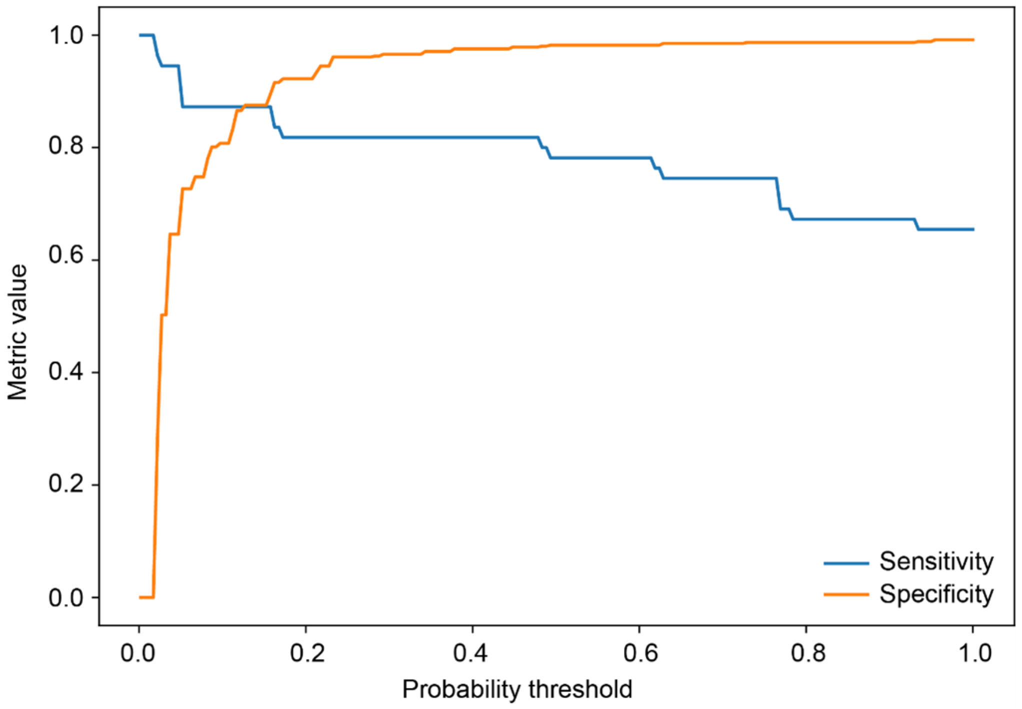

Supplementary Figure S3 Sensitivity-specificity trade-off across thresholds(overall cohort)

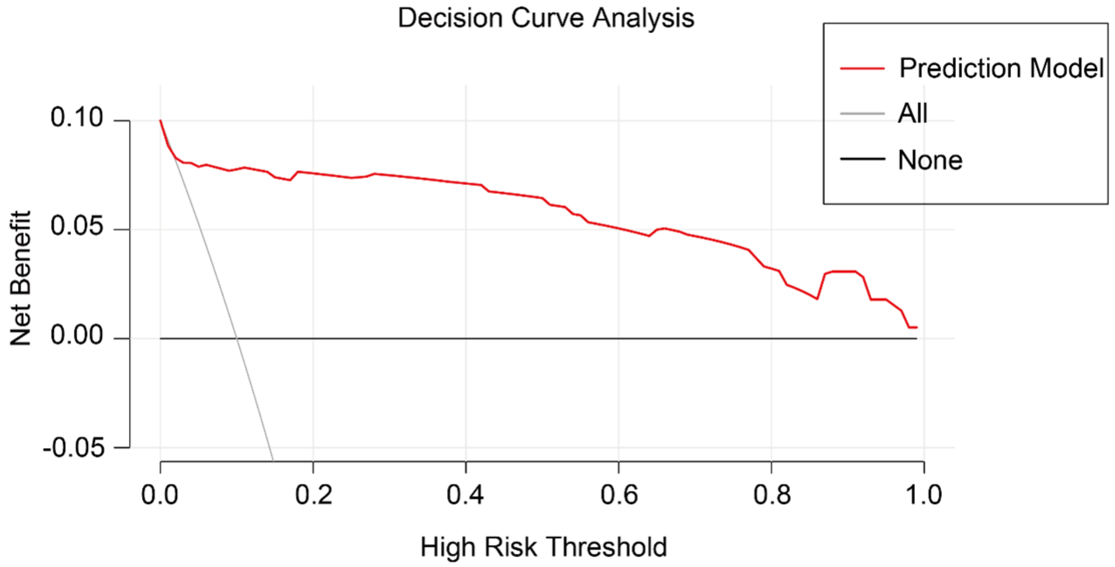

Supplementary Figure S4 Decision curve analysis (DCA) of the nomogram model

STROBE Statement—Checklist of items that should be included in reports of ***cohort studies***

	Item No	Recommendation	Page No
**Title and abstract**	1	(*a*) Indicate the study's design with a commonly used term in the title or the abstract	2-3
		(*b*) Provide in the abstract an informative and balanced summary of what was done and what was found	2-3
**Introduction**			
Background/rationale	2	Explain the scientific background and rationale for the investigation being reported	3
Objectives	3	State specific objectives, including any prespecified hypotheses	3-4
**Methods**			
Study design	4	Present key elements of study design early in the paper	4
Setting	5	Describe the setting, locations, and relevant dates, including periods of recruitment, exposure, follow-up, and data collection	4
Participants	6	(*a*) Give the eligibility criteria, and the sources and methods of selection of participants. Describe methods of follow-up	5
		(*b*) For matched studies, give matching criteria and number of exposed and unexposed	5
Variables	7	Clearly define all outcomes, exposures, predictors, potential confounders, and effect modifiers. Give diagnostic criteria, if applicable	5-6
Data sources/ measurement	8*	For each variable of interest, give sources of data and details of methods of assessment (measurement). Describe comparability of assessment methods if there is more than one group	5-6
Bias	9	Describe any efforts to address potential sources of bias	4-5
Study size	10	Explain how the study size was arrived at	8
Quantitative variables	11	Explain how quantitative variables were handled in the analyses. If applicable, describe which groupings were chosen and why	8
Statistical methods	12	(*a*) Describe all statistical methods, including those used to control for confounding	5-7
		(*b*) Describe any methods used to examine subgroups and interactions	7
		(*c*) Explain how missing data were addressed	7
		(*d*) If applicable, explain how loss to follow-up was addressed	7
		(*e*) Describe any sensitivity analyses	7
**Results**			
Participants	13*	(a) Report numbers of individuals at each stage of study—eg numbers potentially eligible, examined for eligibility, confirmed eligible, included in the study, completing follow-up, and analysed	8
		(b) Give reasons for non-participation at each stage	8
		(c) Consider use of a flow diagram	8

Descriptive data	14*	(a) Give characteristics of study participants (eg demographic, clinical, social) and information on exposures and potential confounders	8-10
		(b) Indicate number of participants with missing data for each variable of interest	8-10
		(c) Summarise follow-up time (eg, average and total amount)	8-10
Outcome data	15*	Report numbers of outcome events or summary measures over time	8-10
Main results	16	(*a*) Give unadjusted estimates and, if applicable, confounder-adjusted estimates and their precision (eg, 95% confidence interval). Make clear which confounders were adjusted for and why they were included	8-10
		(*b*) Report category boundaries when continuous variables were categorized	8-10
		(*c*) If relevant, consider translating estimates of relative risk into absolute risk for a meaningful time period	8-10
Other analyses	17	Report other analyses done—eg analyses of subgroups and interactions, and sensitivity analyses	8-10
**Discussion**			
Key results	18	Summarise key results with reference to study objectives	10-11
Limitations	19	Discuss limitations of the study, taking into account sources of potential bias or imprecision. Discuss both direction and magnitude of any potential bias	13
Interpretation	20	Give a cautious overall interpretation of results considering objectives, limitations, multiplicity of analyses, results from similar studies, and other relevant evidence	13
Generalisability	21	Discuss the generalisability (external validity) of the study results	12
**Other information**			
Funding	22	Give the source of funding and the role of the funders for the present study and, if applicable, for the original study on which the present article is based	15

*Give information separately for exposed and unexposed groups.

Note: An Explanation and Elaboration article discusses each checklist item and gives methodological background and published examples of transparent reporting. The STROBE checklist is best used in conjunction with this article (freely available on the Web sites of PLoS Medicine at http://www.plosmedicine.org/, Annals of Internal Medicine at http://www.annals.org/, and Epidemiology at http://www.epidem.com/). Information on the STROBE Initiative is available at http://www.strobe-statement.org.
